# Effect of Al–Zn Alloy Coating on Corrosion Fatigue Behavior of X80 Riser Steel

**DOI:** 10.3390/ma12091520

**Published:** 2019-05-09

**Authors:** Zhongying Han, Xiaoguang Huang, Zhicheng Yang

**Affiliations:** 1 School of Petroleum Engineering, China University of Petroleum (East China), Qingdao 266580, China; hzy_0218@163.com; 2 College of Pipeline and Civil Engineering, China University of Petroleum (East China), Qingdao 266580, China; 3 Guangzhou University-Tamkang University Joint Research Centre for Engineering Structure Disaster Prevention and Control, Guangzhou University, Guangzhou 510006, China

**Keywords:** corrosion fatigue, arc spray, Al–Zn coating, crack initiation, crack propagation

## Abstract

This paper presents a corrosion fatigue cyclic failure test for X80 steel, which has arc sprayed with an Al–Zn coating in natural seawater under different stress levels. We found that the Al–Zn coating can significantly improve the corrosion fatigue resistance and slow the crack initiation of X80 steel. The effect of the Al–Zn coating on the corrosion fatigue crack initiation is mainly attributed to its physical isolation, cathodic protection and residual prestress while the effect on crack propagation is due to its inhibition of the formation and evolution of secondary cracks. Moreover, according to the test results, a new life prediction model for corrosion fatigue based on the damage evolution law is proposed and the effect of corrosion–fatigue coupling damage in the proposed model is also considered.

## 1. Introduction

As a piece of key equipment for deep-sea oil exploration, the safety and reliability of drilling risers have attracted an increasing amount of attention [1]. The riser always undergoes large reciprocating deformation under the actions of the currents and waves in addition to suffering from the corrosion effect of seawater. Furthermore, corrosion fatigue is one of the main failure modes for these slender flexible structures [2,3,4]. The failure period of corrosion fatigue is generally divided into three stages: Crack nucleation, fatigue crack propagation, and rupture. The first two stages determine the service life [5,6]. Corrosion fatigue failure preferentially initiates from corrosion pits [7,8] due to the anodic dissolution inside the pits accelerated by mechanochemical effects [7] and irreparable damage caused by corrosion products [8]. Although the mechanism of fatigue crack propagation is very complicated and it is difficult to generalize this mechanism, the effect of corrosion on crack propagation is definitely not negligible [9,10,11]. Therefore, the methods for inhibiting corrosion to extend the corrosion fatigue life has been widely adopted, such as surface enhancement by laser [12], low plasticity burnishing [13] and cathodic protection.

Cathodic protection has been widely used for the corrosion protection of metallic materials, which is mainly implemented by a forced current and a sacrificial anode. Compared with a forced current, it is easy to operate a sacrificial anode and it is not affected by the environment. Sacrificial anode protection can be realized by thermally spraying the coating, cold spraying the coating and creating cathodic polarization with sacrificial anodes connected to the specimen, thereby improving the corrosion resistance of the substrate. Aluminum, zinc and their alloys are commonly used as cathodic protection materials due to their relative good corrosion resistance and sufficient negative potential that make them suitable for harsh environments [14]. Simultaneously, the techniques of anodic coating, such as cold spraying, thermal spraying, and hot-dipping spraying, and their potential applications have been greatly promoted. Furthermore, their efficacy - and suitability for different environments have also attracted a wide range of research interests. Diab studied the corrosion and corrosion fatigue behavior of magnesium (3% Al–1% Zn) extrusion with a pure aluminum cold spray coating and found that the pure Al coating provided significant corrosion protection for AZ31B in 5% NaCl fog environment despite the unsatisfactory protection provided against corrosion fatigue [15]. Meanwhile, Al 7075 deposited on AZ31B alloy significantly increased corrosion resistance and lengthened the fatigue life compared to the uncoated specimen in a corrosive environment [16]. Okabe [17] found that the corrosion resistance and corrosion fatigue resistance of steel coated with Zn–Al (–Si and–Mg) and Zn (–Mn,–Cr and–Ni) alloys by a hot-dipping spray improved after the treatment although this depended on the coating conditions. Tachibana [18] compared the influences of a fine Zn and Zn–Al alloy double coating created by hot dipping on the corrosion resistance of steel in coastal areas and found that the life of the Zn–7Al alloy-coated steel was four times longer than that of the Zn–coated steel. Ahnia [19] detected the corrosion reduction of the arc-sprayed aluminum coatings on steel in a simulated marine environment and the tests showed that iron dissolution through the coating decreases with an increase in the annealing temperature. Zhao [20] found that the thermal spraying aluminum coating could significantly improve the corrosion fatigue life of the steel substrate according to the corrosion fatigue crack propagation experiments of aluminum coated X80 steel in 3.5 wt % NaCl solution. In addition to the aluminum and Zinc coating, Zinc–chromium coating was also used for the corrosion and corrosion fatigue protection of mild steel [21]. Villalobos-Gutiérrez et al. [22] evaluated the effect of WC–10Co–4Cr thermal spraying coating by high-velocity oxygen fuel deposition (HVOF) on the fatigue and corrosion properties of AA6063–T6 aluminum alloy. The results showed that the HVOF thermal spray process gave rise to significant gains in fatigue life in comparison with the uncoated substrate when testing was carried out both in air and in a 3 wt % NaCl solution. Among the three anodic coating techniques, thermal spraying is the most highly developed and widely applied in marine engineering. Long-time practical applications also showed that thermal spraying coatings could effectively protect marine structures from corrosion [23,24,25].

Anodic coatings protect marine steel from corrosion and corrosion fatigue by physical isolation and cathodic protection. Therefore, the spraying qualities, such as the porosity and electrochemical activity of coatings, significantly determine the protection effect [26,27]. Meanwhile, most marine structures, such as deepwater risers, are subject to a severe environment and random bending loads [28]. The effects of anodic coatings on corrosion fatigue behavior are essential for the applications in marine structures. Furthermore, the mechanism related to the improvement of corrosion fatigue behavior due to anodic coatings requires more indepth study and experimental support in view of the material and environment dependence of corrosion fatigue. In this paper, the corrosion fatigue tests of the X80 riser steel in natural seawater are carried out for studying the effect of Al–Zn coating by arc spraying on the corrosion fatigue life. Furthermore, the mechanism related to the coating -inhibiting the crack initiation and propagation is studied by fracture analysis technology.

## 2. Corrosion Fatigue Test

**Figure 1:**
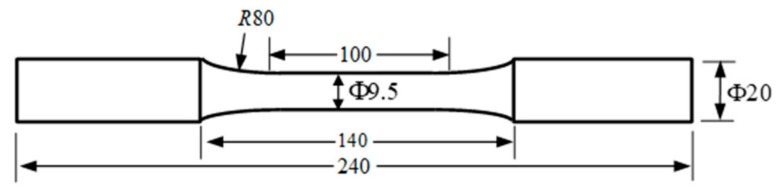
Dimension of smooth cylindrical specimen (mm).

The X80 steel used in the test contained (wt %) 0.048% C, 0.195% Si, 1.717% Mn, 0.012% P, 0.002% S, 0.219% Cr, 0.184% Mo, 0.268% Ni, 0.023% Al and the remainder was Fe. The yield strength *σ*_s_ and tensile strength *σ*_b_ of X80 steel were 680 MPa and 710 MPa, respectively, which indicated that the X80 steel had a better ability to resist deformation. The cylindrical specimens were obtained from an X80 steel bar (Φ24 mm) by wire-cutting and fine grinding for smoothness as shown in Figure 1.

**Figure 2:**
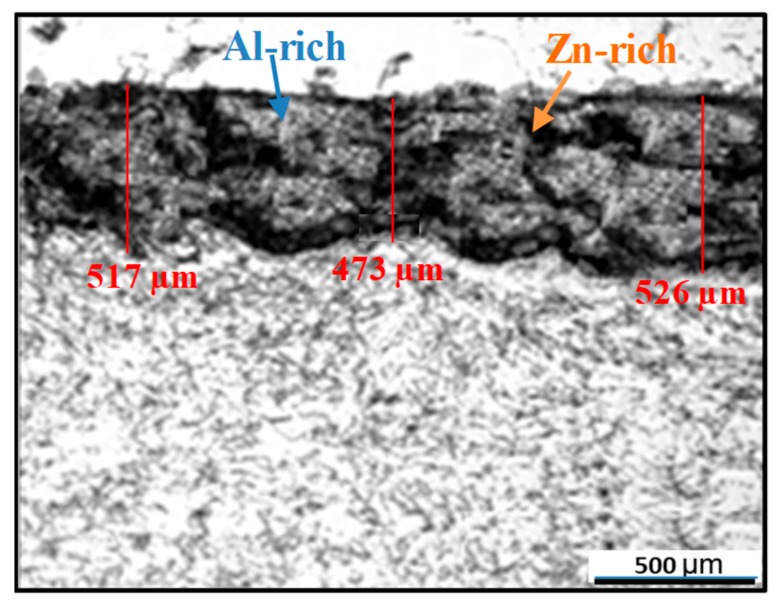
Cross-section microstructure of coated specimen.

Before the arc spraying, the rust on specimens needs to be removed. The surface of specimens was blasted by corundum at a pressure and distance of 0.6 MPa and 150 mm, respectively, to achieve the surface roughness of 50–80 µm. After that, the Al–Zn alloy wire (85% Al, 15% Zn) with a 2 mm diameter was used in a CMD–AS1620 arc spraying system (Xindi, Beijing, - China), while the distance between the nozzle and the substrate was kept at 150 mm. During the spraying, the stagnation pressure and the wire feed rate used in the spraying system were set as 0.7 MPa and 10 cm/min, respectively, and the coating thickness was kept at 500 ± 30 µm by finely adjusting the process parameters of the spraying system. The hardness measurement at a load of 0.98 N shows that the average Vickers hardness near the surface of the coated sample is 147.2 ± 3.6 HV, which is obviously lower than that of the uncoated sample (241.3 ± 6.3 HV). An IPRE-SR200 surface roughness tester (Purui, Shenzhen, Guangzhou, -China) is utilized to measure the coated surface, and the roughness results measured from different positions are taken the average to eliminate the influence of error as much as possible. The measured surface roughness of the coated sample is 63.5 ± 8.4 µm. The microstructure of the coated specimen observed under an optical microscope is shown in Figure 2. The coating thickness meets the pre-requirement according to the thickness measurement at three different locations. It can be noted that the coating represents a pseudo two-phase alloy structure, within which the grey–white part is the Al-rich phase and the grey-black part is the Zn-rich phase. Furthermore, grey–white phase and grey–dark phase of the coatings stack alternately on the substrate in a wavy form. This is attributed to the fact that the Al–Zn alloy undergoes a crystallization process from melting to cooling during the preparation of the coating and the rapid solidification process prevents Al–Zn from completely melting, which results in the presence of the two-phase structure.

**Figure 3:**
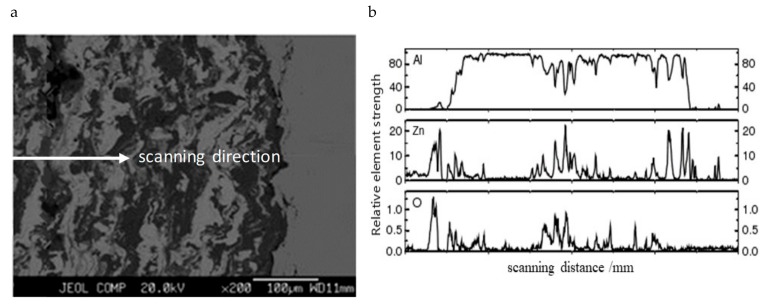
EMPA analysis of (**a**) scanned image and (**b**) analysis results.

To ensure that the Al–Zn alloy was well coated on the specimens, the microstructures and elemental compositions of the coating were detected by observing the sectioning microstructures and applying the electron microprobe analysis (EMPA) method (JEOL, Tokyo, Japan). The corresponding results are shown in Figure 3. The electron microprobe scanned along the thickness of the coating (Figure 3a) and showed the content distribution of Al and Zn (Figure 3b) in the coating. However, there was a higher oxygen content somewhere in the coating along its thickness. This could be the result of aluminum oxide inclusion in the coating when spraying.

**Figure 4:**
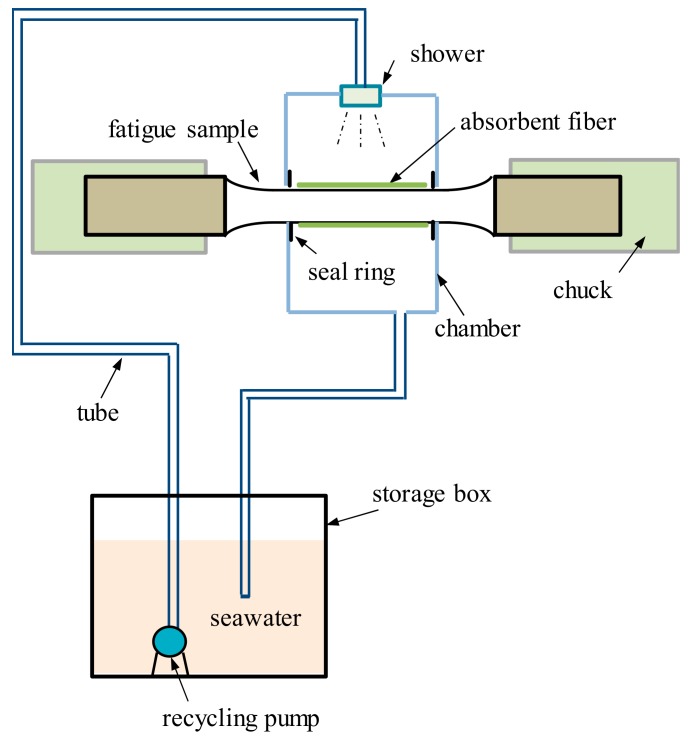
Setup of circulation device of corrosion solution matching with fatigue testing machine.

For the facilitation of the corrosion fatigue failure test, a special seawater circulation system (Figure 4) was designed to ensure there was a good interaction between the specimen and seawater during the fatigue test. The seawater circulation system was composed of a storage box, a chamber, a tube, a recycling pump, and a shower. The seawater was driven by the pump and sprays on the specimen in the chamber before being collected by the storage box. The test section of the specimen was wrapped in an absorbent fiber for good infiltration into the seawater. Finally, the corrosion fatigue failure tests were carried out by a cardan low-frequency rotating bending fatigue testing machine and four stress amplitudes (204 MPa, 272 MPa, 408 MPa, and 544 MPa) were used while the load frequencies for each stress were set as 0.5 Hz and 2 Hz, respectively.

## 3. Results and Discussion

### 3.1. Coating on Corrosion Fatigue Life

**Figure 5:**
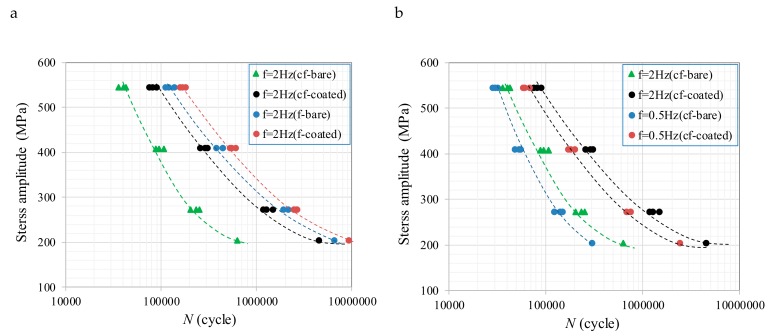
Comparison between fatigue and corrosion fatigue life in number of failure cycles: (**a**) Fatigue and corrosion fatigue life at *f* = 2 Hz and (**b**) load frequency on corrosion fatigue life.

Figure 5 shows the effect of stress amplitudes, load frequencies, and coating on the fatigue life in a number of failure cycles. Figure 5a shows that when the load frequency is assigned to 2 Hz, the fatigue life of both coated and bare X80 steel is shorter in the presence of corrosion. It also can be noted that the corrosion fatigue life of the coated X80 steel is longer than that without coating and it decreases as the stress amplitude increases. From Figure 5b, the number of corrosion fatigue failure cycles of the X80 steels is relatively related to the load frequency and becomes smaller when there is a lower load frequency. The effect of the coating on fatigue is not as remarkable as that on corrosion fatigue due to the surface roughness being critical to fatigue crack initiation without the interaction of the corrosive environment. Arc spraying slightly increases the size of the specimen and improves the prestress state on the surface. However, the roughness of the coating surface makes it difficult to achieve a machining accuracy of the uncoated sample, and to some extent, this accelerates the crack initiation at the surface.

### 3.2. Mechanism of Coating Improving Corrosion Fatigue Life

**Figure 6:**
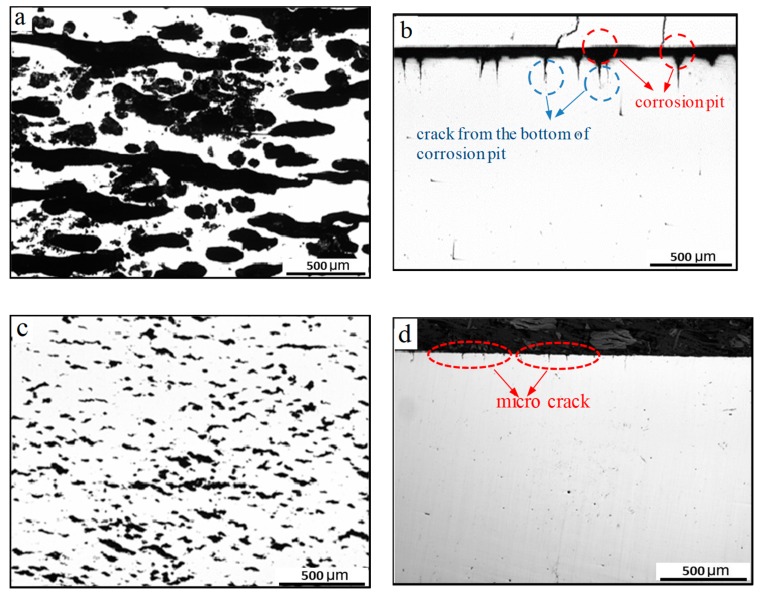
Metallography of bare specimen at the surface and cross-section after corrosion fatigue at 2 Hz: (**a**,**b**) 276 MPa and (**c**,**d**) 552 MPa.

Figure 6a,b shows the metallographic morphologies of corrosion and crack at the surface and cross-section of the bare specimen under low stress (276 MPa) after 100,000 cycles at a frequency of 2 Hz. From Figure 6a,b, it can be observed that a significant number of corrosion pits and some conspicuous cracks in the bottom of the corrosion pit appear at the surface when the stress amplitude adopted in the test is 276 MPa. However, when the stress amplitude increases to 522 MPa, there are no obvious corrosion pits but numerous tiny cracks are formed at the surface and cross-section (Figure 6c,d) after 15,000 cycles. This could be that the specimen subjected to a higher stress amplitude has a shorter fatigue life and the contact time between the specimen and corrosion solution is limited. There are no obvious corrosion pits forming at the surface of the specimen.

Obviously, the contribution of the corrosion to crack nucleation differs under different stress levels. Stress softening is a common phenomenon at low cycle fatigue of the X80 steel due to its high yield ratio. Plastic deformation caused by cyclic softening results in the regional material being easy to slip. This forms the resident slip zone, resulting in a local preferential dissolution of material and fatigue crack initiation [29]. As a result, the bare specimens are vulnerable to fractures under high-stress conditions due to the existence of resident slip bands. Meanwhile, crack propagation also causes stress release, which makes it difficult for the cracks in other regions to form or expand. This further explains why the cracks under high-stress conditions are shorter and smaller from metallographic pictures.

**Figure 7:**
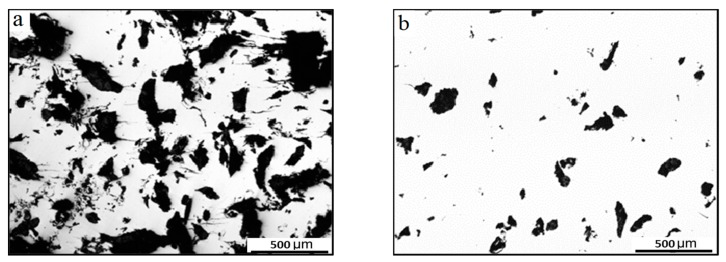
Surface metallographic photographs of corrosion fatigue formation of coated specimen: (**a**) 276 MPa after 100,000 cycles and (**b**) 552 MPa after 50,000 cycles.

Figure 7 shows the surface metallographic morphology of the coated specimen after certain cycles. Figure 7a shows the surface morphology under low-stress conditions (276 MPa) after 100,000 cycles at a frequency of 2 Hz. It can be seen that a significant number of corrosion pits in black appear on the coating surface, while the corrosion is significantly less than that in Figure 6a. Figure 7b shows the surface morphology under high-stress conditions (552 MPa) after 50,000 cycles. At high-stress levels, no obvious corrosion pits and corrosion fatigue cracks appear on the surface of the specimen.

**Figure 8:**
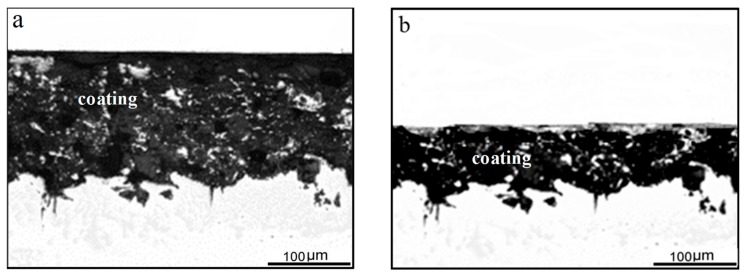
Metallographic microstructure of coatings: (**a**) After 300,000 cycles and (**b**) after 400,000 cycles.

Figure 8 depicts the microstructural changes in the coating after 300,000 and 400,000 cycles. It can be seen that the thickness of the layer gradually reduces and the size of the pore in the coating also increases. By comparing the crack initiation and fatigue life of bare and coated specimens, it is not hard to determine that the arc spraying significantly inhibits the initiation of corrosion fatigue cracks. The effects of the coating on fatigue crack initiation mainly lie in the following aspects, i.e., physical isolation, cathodic protection, and residual stress at the surface made by sandblasting. The coating acts as an isolating layer between the substrate and seawater until it consumes too much to achieve complete physical isolation. The self-corrosion potentials of aluminum and zinc are both about −1.0 V, and that of X80 steel before sandblasting is about −0.6 V in the seawater vs. a saturated calomel electrode (SCE). Therefore, the coating provides the cathodic protection of the X80 as it acts as a sacrificial anode during the corrosion fatigue process. Sandblasting before the arc spraying can cause certain compressive stress on the surface, and a certain plastic deformation will occur on the surface of the specimen when the compressive stress exceeds the yield strength of the material. It is believed that the compressive stress and compression plastic deformation can prevent fatigue crack initiation [30,31]. The rheological microstructure at the surface is protected from the isolation of the coating and the effect of residual compressive stress and plastic deformation on the corrosion fatigue crack initiation are brought into full play when the coating loses its complete isolation of the substrate from the seawater.

**Figure 9:**
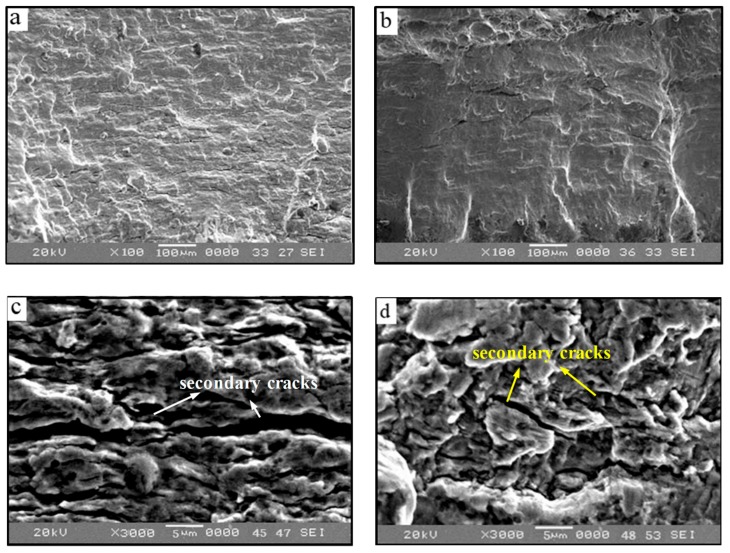
Fracture morphology of crack propagation zone: (**a**,**c**) Corrosion fatigue striations and secondary cracks of bare specimen; and (**b**,**d**) corrosion fatigue striations and secondary cracks of coated specimen.

The most distinctive feature of crack propagation is the formation of fatigue striations. The secondary cracks are closely related to the crack propagation and formation of corrosion fatigue striations. Furthermore, the effect of the coating on fatigue crack propagation can be explained by the formation and evolution of secondary cracks. Therefore, the mechanism of the Al–Zn coating on the corrosion fatigue crack of X80 steel can be explained by comparing the formation of the corrosion fatigue striation of bare steel and coated steel. Figure 9a,b shows the morphologies of the fracture propagation zone of bare and coated specimens under high-stress conditions. There are innumerable secondary cracks and corrosion products on the fracture surface of bare specimens, while the corrosion and the number of secondary cracks are significantly less on the fracture surface of coated specimens. The secondary crack striations of the bare and coated steels are observed more carefully in Figure 9c,d. We found that the direction of the secondary crack striations is almost the same as that of the corrosion fatigue striations. The main difference is that the secondary crack striation on the surface of bare specimens is deeper than that on the coated specimens. Meanwhile, the fatigue striations of bare specimens are less clear as they are covered in a layer of corrosion products. It can be concluded that the existence of corrosion products aggravates the irreversibility of the grain boundary slip, which leads to the accumulation of irreversible damage and promotes the propagation of secondary cracks. Conversely, the coating provides cathodic protection and greatly reduces the corrosion and irreversible slip at the crack tip. Therefore, the corrosion fatigue damage cannot easily accumulate and the crack propagation is partly inhibited.

## 4. Discussion

According to the damage mechanics, the corrosion fatigue is regarded as a process that results in damage initiating and accumulating inside the material under the coupling action of the corrosion environment and cyclic loading. Hence, the total evolution law of corrosion fatigue damage can be expressed as follows [32]:

(1) $$\frac{dD}{dt}=\frac{dD_{c}}{dt}+\frac{dD_{\mathrm{scc}}}{dt}+T_{0}\frac{dD_{f}}{dt}$$

where *D*, *D*_c_, *D*_scc_ and *D*_f_ are the corrosion fatigue damage, corrosion damage, stress corrosion damage induced by average stress, and fatigue damage caused by stress amplitude, respectively, *t* is time, *N* is the number of cycles, and *T*_0_ is the period of fatigue loading.

If the evolution law of corrosion damage and corrosion stress damage is adopted as follows:

(2) $$\frac{dD_{c}}{dt}=\frac{c_{0}+c_{1}{\left(\sigma_{0}+\sigma_{a}\right)}^{\alpha }}{{\left(1-D\right)}^{m}}$$

(3) $$\frac{dD_{\mathrm{scc}}}{dt}=c_{\mathrm{scc}}{\left(\frac{\sigma_{0}}{1-D}\right)}^{\zeta }H_{0}$$

where *c*_0_ is the damage accumulating factor in a stress-free state, *m* is the accumulating index of corrosion damage, *σ*_0_ and *σ*_a_ are the average stress and stress amplitude, respectively, *c*_1_(*σ*_0_ + *σ*_a_)*^α^* is the accelerating effect of stress on corrosion damage, *α* and *ζ* are constants related to the material, *c*_scc_ is the accumulating factor of stress corrosion damage, *σ*_scc_ is the nominal threshold stress of stress corrosion, and *H*_m_ is the activation function of stress corrosion damage, which can be expressed as:

(4) $$H_{0}=\left\{\begin{array}{ll} 1-{\left(\frac{\left(1-D\right)\sigma_{\mathrm{scc}}}{\sigma_{0}}\right)}^{\gamma } & \mathrm{when}\frac{\sigma_{0}}{1-D}>\sigma_{\mathrm{scc}}\\ 0 & \mathrm{when}\frac{\sigma_{0}}{1-D}\leq \sigma_{\mathrm{scc}} \end{array}\right.$$

where *γ* is a material constant.

When only the influence of cyclic stress amplitude is considered in the above-mentioned fatigue damage model, the fatigue damage per cycle can be simplified as follows [33]:

(5) $$\frac{dD_{f}}{dN}={\left(1-D\right)}^{-\mu }{\left(\frac{\sigma_{a}}{M\left(\sigma_{0}\right)}\right)}^{\xi }$$

where *μ* and *ξ* are the experimental constants and *M*(*σ*_0_) is a material parameter that is related to the average stress. Therefore, the damage evolution law of corrosion fatigue can be written as:

(6) $$\frac{dD}{dN}=\frac{c_{0}+c_{1}{\left(\sigma_{0}+\sigma_{a}\right)}^{\alpha }}{{\left(1-D\right)}^{m}}T_{0}+c_{\mathrm{scc}}{\left(\frac{\sigma_{0}}{1-D}\right)}^{\zeta }T_{0}H_{0}+{\left(1-D\right)}^{-\mu }{\left(\frac{\sigma_{a}}{M\left(\sigma_{0}\right)}\right)}^{\xi }$$

For the symmetrical rotating bending corrosion fatigue, the damage law can be simplified as:

(7) $$\frac{dD}{dN}=\frac{c_{0}}{{\left(1-D\right)}^{m}}T_{0}+{\left(1-D\right)}^{-\mu }{\left(\frac{\sigma_{a}}{M\left(\sigma_{0}\right)}\right)}^{\xi }$$

By integrating Equation (7), the corrosion fatigue life *N* expressed by damage evolution is obtained as:

(8) $$N=\int_{D_{0}}^{D_{c}}\frac{dD}{c_{0}T_{0}{\left(1-D\right)}^{-m}+{\left(1-D\right)}^{-\mu }{\left(\frac{\sigma_{a}}{M\left(0\right)}\right)}^{\xi }}$$

where *D*_0_ is the initial damage that is usually taken as 0 and *D*_c_ is the critical damage, which is taken as one for simplicity.

**Figure 10:**
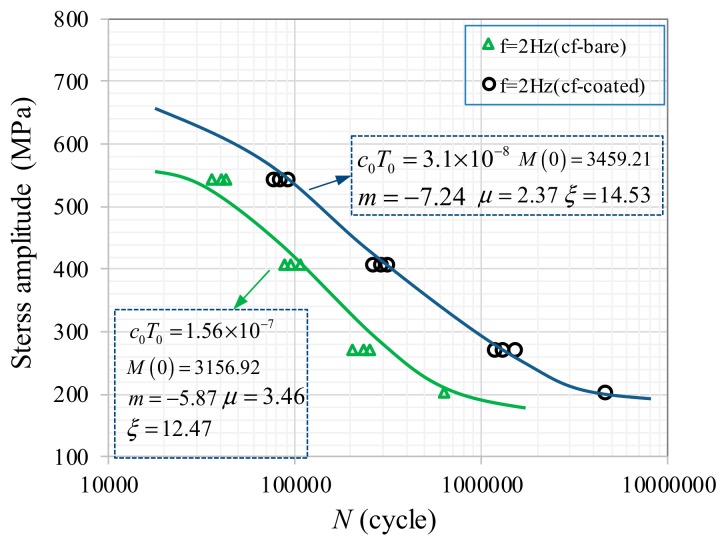
Regression results in corrosion fatigue damage evolution law.

The parameters *μ*, *ξ,* and *M*(*σ*_0_) can be determined according to the fatigue test. The evolution life of the corrosion fatigue damage is determined using Equation (8) by adopting d*D* = Δ*D* = 0.001. Figure 10 shows the regression results for the corrosion fatigue of the bare and coated samples at *f* = 2 Hz. It can be noted that there is good consistency between the test result and predicted results, which indicates that the prediction model can provide reasonable predictions for corrosion fatigue life.

## 5. Conclusions

This paper conducted an experimental investigation of the corrosion fatigue behavior of X80 steel with an Al–Zn coating. The effects of stress amplitude, load frequency and the presence of the coating on the corrosion fatigue life of X80 steels were studied. A prediction model for predicting the corrosion fatigue life was verified by the test results. We found that the Al–Zn coating can significantly improve the corrosion fatigue life of X80 steel. The load frequency and the stress amplitude used in the test significantly influence the corrosion fatigue life of X80 steel. The coating on X80 steel can inhibit the initiation and propagation of the corrosion fatigue crack compared to X80 steel without a coating. The effect from arc spraying the Al–Zn coating on fatigue crack initiation is mainly related to the physical isolation, cathodic protection. and residual stress at surface made by sandblasting. The effect of Al–Zn coating on crack propagation is mainly reflected in the inhibition of the formation and evolution of secondary cracks. A new damage evolution law for corrosion fatigue is also proposed to consider the accumulation mechanism of corrosion-fatigue coupling damage, which can provide reasonable predictions for corrosion fatigue life compared to the test results.
